# Cost-effectiveness of dialectical behavioural therapy versus treatment as usual for autism with suicidal behaviours: single-blind randomised controlled trial

**DOI:** 10.1007/s10198-025-01794-3

**Published:** 2025-05-31

**Authors:** Anne Huntjens, Filip Smit, L. M. C. van den Bosch, Ad Kerkhof, Bram Sizoo, Mark van der Gaag

**Affiliations:** 1 Department of Clinical Psychology, VU University and Amsterdam Public Mental Health Research Institute, Amsterdam, Netherlands; 2 Department of Mark van der Gaag Research Centre, Parnassia Psychiatric Institute, The Hague, Netherlands; 3 Department of Epidemiology and Biostatistics, Amsterdam University Medical Centers, Amsterdam, Netherlands; 4 Independent Researcher, Dialexis, Nijmegen, Netherlands; 5 Department of Clinical Psychology, University of Amsterdam, Amsterdam, Netherlands; 6 Department Clinical Psychology, Vrije Universiteit Amsterdam, De Boelelaan 1105, 1081 HV Amsterdam, The Netherlands

**Keywords:** Autism, Dialectical behaviour therapy, Suicidal behaviours, Cost-effectiveness, Economic evaluation, H51 - H52

## Abstract

**Objective:**

This study evaluated the cost-effectiveness of dialectical behaviour therapy (DBT) compared to treatment as usual (TAU) for autistic adults with suicidal behaviours*.*

**Method:**

In a randomised controlled trial, 123 autistic outpatients were assessed over 12 months. Healthcare costs and societal costs were calculated in accordance with the Dutch standard. Outcomes were quality-adjusted life years (QALYs) and treatment response, defined as a reduction of at least 50% in symptoms of suicidal ideation from t_0_—t_12_ as measured by the Suicidal Ideation Attributes Scale (SIDAS), plus achieving SIDAS < 20 at t_12_ (i.e. below the clinical threshold).

**Results:**

From the healthcare perspective, DBT cost €371 less than TAU while gaining an additional 0.184 QALYs, with a 64% likelihood of being the dominant treatment option. From the societal perspective, DBT has higher costs than TAU by €232 per QALY gained, which can be considered cost-effective given a willingness-to-pay of €50,000 per QALY. DBT also showed better treatment response rates, with less suicidal ideation, at lower costs than TAU. Sensitivity analyses supported these findings.

**Conclusion:**

DBT is a novel treatment for autistic adults with suicidality. It fills a significant treatment gap in lieu of any evidence-based alternative for this population. DBT reduces suicidality, enhances quality of life and is cost-effective across healthcare and societal perspectives, encouraging broader adoption. Future research should assess DBT’s long-term impacts and its transferability to other countries and map pathways towards upscaled implementation.

**Supplementary Information:**

The online version contains supplementary material available at 10.1007/s10198-025-01794-3.

## Introduction

Autism Spectrum Disorder (ASD) is a lifelong neurodevelopmental disorder characterised by a persistent vulnerability for deficits in social communication and interaction and limited repetitive patterns of behaviour, interests, and activities. ASD affects 1.1% of the population [1, 2], and is often accompanied by psychiatric comorbidities presenting additional challenges for independent living and employment [1–6]. Even more concerning, autistic adults face an elevated risk of suicidal ideation (SI) and suicide attempts (SA), with a lifetime prevalence of 37.2% for SI and 13.4% for SA [7].

ASD places a significant disease burden on the population, resulting in considerable economic costs for both society and healthcare systems [8–10]. The primary drivers of these costs include healthcare expenses and productivity losses. In essence, autism carries a vast financial burden across multiple areas, leading to considerable overall lifetime costs related to the disorder [8–10].

Dialectical Behaviour Therapy (DBT), known for effectively addressing suicidal behaviours across various psychiatric conditions and reducing the number of hospital admissions [11]. DBT has shown effectiveness in autistic adults experiencing suicidal behaviours [12, 13], emerging as a novel intervention that fills a significant treatment gap where no evidence-based treatment existed previously.

DBT is a widely recognised, evidence-based treatment known for its effectiveness in addressing suicidal behaviour across various psychiatric conditions through therapy, group skills training, and phone consultations as needed [14]. Sessions follow a set sequence based on treatment priorities by first addressing life-threatening and therapy-interfering behaviours, then improving quality-of-life and skill generalisation. DBT teaches four key skills: mindfulness, distress tolerance, emotion regulation, and interpersonal effectiveness, with weekly practice.

Recent research has demonstrated the effectiveness of DBT as a treatment for suicidal behaviour in autistic individuals [12, 13]. This paper aims to explore the cost-utility and cost-effectiveness of a DBT intervention for improving health-related quality of life and reducing suicidal ideation in a multicentre randomised controlled trial [12, 13].

## Materials and methods

### Design and participants

The full details of the study are reported elsewhere [12, 13]. In brief, the study was designed as a pragmatic, single-blind, randomised, controlled multicentre trial with two parallel arms, DBT and TAU, measuring costs and effects at baseline (t_0_), 6 months post-baseline (t_6_), and 12 months follow-up (t_12_).

Participants were recruited from six Dutch mental health services between September 2018 and December 2022. Eligible participants were adult outpatients in care for ASD with suicidal behaviours who had received an ASD diagnosis from experienced clinicians, following national guidelines prior to inclusion [15]. The diagnostic status was confirmed by the Autism-Spectrum Quotient (AQ-short) [16]. Suicidal behaviour was assessed by the Suicidal Ideation Attributes Scale (SIDAS score ≥ 21) [17] and the Lifetime Parasuicide Count (LPC) [18]. Exclusion criteria were (1) IQ < 80 assessed with WAIS-IV only if baseline testing was difficult due to intellectual deficits [19]; (2) addiction to drugs and need for clinical detoxification; and (3) insufficient command of the Dutch language.

Eligible participants (*N* = 123) signed an informed consent form and were randomised to either DBT (*N* = 63*)* or TAU (*N* = 60). The independent randomisation office of the Parnassia Psychiatric Institute randomised participants using www.randomizer.org. Assessors were masked for randomised status. The study was registered at isrctn.com (ISRCTN96632579) and approved by the Medical Ethics Committee of the Vrije Universiteit Medical Centre (NL59497.029.17).

### Interventions

#### DBT

DBT was offered as a time-limited intervention for a maximum of six months. The intervention was based on the four components of comprehensive DBT, combining a) weekly 45-min individual cognitive-behavioural psychotherapy sessions with the primary therapist, b) weekly 2 h15 skills training group, c) if needed, access to telephone coaching with their individual therapist, and d) weekly 1-h therapist consultation [14]. Participants randomised to the DBT group received two pretreatment sessions with their assigned primary DBT therapist, preparing them for the demands and expectations of DBT. This process involved familiarising clients with the therapy's structure, components, and goals. Individual psychotherapy takes place weekly and primarily focuses on motivational issues, including the motivation to stay alive and remain in treatment. The group skill training in this study, adapted from Neacsiu’s (2014) emotion regulation skill training [20], featured two weekly group skill training. This format provided extensive opportunities for feedback, rehearsal, and practice, ultimately leading to enhanced skill learning tailored to the needs of autistic adults [21]. During the follow-up period, participants continued to utilise various health services provided by their general practitioners, social services, and other healthcare providers.

#### TAU

The control group received treatment as usual (TAU), involving at least weekly 45-min sessions with a psychotherapist or social worker. TAU encompassed any common form of treatment for suicidal behaviour in autism within the Dutch mental health system, ensuring a realistic reflection of current clinical practice in terms of form and intensity (e.g., psycho-education, social skills training, emotion regulation therapy, cognitive-behavioural therapy, trauma therapy, delivered in both group and individual formats, either independently or concurrently). TAU was not a time-limited intervention but lasted as long as the client and therapist deemed necessary.

#### Therapists

Treatment was provided by mental health professionals working at specialised services for autism. All professionals in both conditions had significant experience in treating autistic individuals and were either psychologists, psychiatrists, or social psychiatric nurses.

### Outcomes

#### Quality-adjusted life years

Quality-adjusted life years (QALYs) were the primary outcome in the cost-utility analysis (CUA). QALYs were computed from participants’ responses on the EQ-5D-3L questionnaire [22] at (t_0_), (t_6_), and follow-up (t_12_). The EQ-5D-3L has five domains (mobility, self-care, usual activities, pain/discomfort, and anxiety/depression), and each dimension has three levels (no problem, some problems, severe problems) such that a total of 3^5^ = 243 health states can be defined. The EQ-5D-3L health states were converted into utilities (quality of life weights) with the Dutch tariff [23]. The utilities are anchored at 0 (death) and 1 (full health), but negative utility scores can occur for health states regarded as worse than death. The utilities were obtained at three measurement points, and QALY gains over the full 12 months of follow-up time were computed using the area under the curve method.

#### Treatment response

Effects in the cost-effectiveness (CEA) were measured in terms of a reduction in suicidal ideation over 12 months using the Suicidal Ideation Attributes Scale, SIDAS [17]. SIDAS comprises five items, each targeting an attribute of suicidal thoughts: frequency, controllability, closeness to attempt, level of distress associated with the thoughts, and impact on daily functioning. Scores of ≥ 21 indicate an elevated risk of suicidal ideation, with a higher score indicating more severe suicidal ideation. For clinical and health-economic interpretation, the SIDAS was converted into a ‘Treatment Response’ outcome defined as a reduction of SIDAS symptoms by at least 50% in the t_0_ – t_12_ time interval plus having a SIDAS score lower than 20 at t_12_, i.e. below the SIDAS threshold for suicidality.

#### Resource use and costing

Healthcare use was assessed using the Trimbos Institute and Institute of Medical Technology Assessment Questionnaire for Costs Associated with Psychiatric Illness (TiC-P) questionnaire, a reliable measure for trial-based economic evaluations in Dutch healthcare [24, 25]. Data were collected at t_0_, t_6_, and t_12,_ with participants reporting healthcare usage and medication use over a 3-month recall period.

Healthcare costs were calculated by multiplying healthcare units (e.g. GP visits, sessions with a psychologist, hospital stays) with their standard unit cost price for the year 2023 as reported in the latest Dutch Guideline for costing and economic evaluations in healthcare [26] (Online Resource Table S1). The medication cost per standard daily dose was obtained from the Dutch Farmacotherapeutisch Kompas [27] and these were multiplied by the number of prescription days and increased by adding the pharmacist's dispensing fee of €7 per 90-day prescription period (Online Resource Table S1).

Patient and family costs stem from travel expenses related to healthcare visits and informal care from the patient's family. Travel costs were estimated using average distance (in km) between any address and health services multiplied by the per km price of using public transport(Online Resource Table S2). Informal care was valued at €18,80 per hour, based on the replacement cost method from the Dutch Guideline for costing and economic evaluations in healthcare [26] (Online Resource Table S1).

Productivity losses arise from absenteeism (work loss days) and presenteeism (days worked less productively while at work) [28]. The latter was estimated by multiplying the reported number of hours worked less productive by a factor reflecting the level of inefficiency during those hours. The total costs attributed to productivity losses were determined by multiplying the number of hours of work lost by €39,88 in paid work and €18,80 in unpaid work based on the method from the Dutch Guideline for costing [26] (Online Resource Table S1).

Supervision costs for therapists providing DBT were included in the analysis as part of healthcare costs. Supervision is essential for maintaining a well-supported and effective treatment program. Supervision costs were calculated based on 16 sessions totalling 12 h annually at €123 per 45-min session, shared among a team of six therapists, each serving an average of 10 patients. This resulted in an annual supervision cost of €32,80 per patient.

Societal costs were the sum of healthcare, patient and family costs, and costs stemming from productivity losses.

### Analysis

The health-economic evaluation was conducted in accordance with the Consolidated Health Economic Evaluation Reporting Standards (CHEERS) guidelines for economic evaluations [29] (Online Resource Table S3). Costs were reported in Euro (€) from the Netherlands for the reference year 2023, when €1.00 had a purchasing power equal to US$1.7299 [30]. Since the trial’s follow-up time did not exceed 12 months, no discounting was applied to costs and effects. The economic evaluation was conducted from both the healthcare and societal perspective.

With *N* = 64 per trial arm, the study was only powered to detect a standardised mean difference of d ≥ 0.50 as statistically significant at a ≤ 0.05 and b ≥ 0.80 on the central clinical outcome (SIDAS) but was not powered to test cost difference. Therefore, inferences about between-group cost and effect differences and incremental cost-effectiveness ratios will not be based on statistical hypothesis testing, but on a probabilistic medical decision-making approach using 2,500 bootstrap replications.

Data missing due to study dropout were imputed using regression imputation for intention-to-treat analysis. Regression imputation is replacing missing values by their predicted values as obtained under a regression model where two types of predictors where used: 1) predictors of the outcome Y, and 2) predictors of missingness, M. The former set of predictors is used to get precise estimates of Y, and the latter is used to meet the MAR assumption when missingness is not completely random but correlated with the demographic, psychological or economic features of the participants. This approach is recommended by Demirtas (2004) [31]. For individuals who died by suicide, QALY was set at 0 (i.e.,"death") and treatment response also at 0 (i.e. “failure”). Cumulative costs and QALY gains over the one-year follow-up time of the trial were obtained through area under the curve method between t_0_, t_6_, and t_12_. Although baseline cost differences between groups were not statistically adjusted for using baseline costs as a covariate, this was deemed acceptable given the focus on within-subject changes in costs and QALYs over time. As such, the potential impact of baseline cost imbalances on between-group comparisons was considered limited. Seemingly unrelated regression equations (SURE) models were used, which were bootstrapped 2,500 times, to simultaneously evaluate between-group cost and effect differences and plot the simulated incremental cost-effectiveness ratios (ICERs) on a cost-effectiveness plane. The ICER plane is divided into four quadrants. If the bulk (> 50%) of simulated ICERs can be found in the Southeast (SE) quadrant, then it is understood that DBT produces more health for less cost than TAU. From a cost-effectiveness perspective, this is the most favourable outcome. The intervention is then said to be ‘dominant’ because it is the preferred treatment option.

If the bulk of simulated ICERs is concentrated in the North-East (NE) quadrant, it indicates that more health is gained by the intervention, albeit at additional costs. This ‘pay more, get more’ rule is a common outcome in health-economic evaluation, and decision-makers must then decide if paying more for gaining more health is acceptable. As a tool for decision-makers, cost-effectiveness acceptability curves (CEACs) were generated. CEACs assess the probability of the intervention being cost-effective across a range of willingness-to-pay ceilings for obtaining one additional QALY or one additional treatment responder. In the Netherlands, the willingness-to-pay ceiling for gaining one QALY ranges from €20,000 to €80,000, depending on the severity of the condition [32]. Although ASD with suicidality might be considered a severe condition, we applied the conventional WTP threshold of €50,000 per QALY in this analysis, because a disability weight for ASD with suicidality is not reported in the literature [33].

All analyses were conducted with Stata 17.0 (StataCorp, 2021).

### Sensitivity analysis

In a pre-planned sensitivity analysis, the base-case analysis was repeated using last observation carried forward (LOCF) imputation to assess the robustness of the results under different imputation strategies [12].

## Results

### Sample

At six Dutch mental health centres, 123 outpatients (aged 18–65 years) with DSM-5 diagnosed autism spectrum disorder and suicidal behaviours were randomly assigned to either dialectical behaviour therapy (DBT) (*n* = 63) or treatment as usual (TAU) (*n* = 60) (Online Resource Fig. S1).

Dropout from the study was kept to a minimum, with 6 participants (9.5%) in the DBT condition and 5 (8.3%) in TAU having missing values at t_12_. The attrition rates were not statistically different across the conditions (c^2^ = 0.05; *df* = 1; *p* = 0.817). In the TAU group, 2 participants died by suicide. Overall, 14 severe adverse events were reported: 9 (15.0%) in TAU, including both suicides and 5 in DBT (7.9%) (for full details, see [13]). The medical-ethical committee considered these adverse events unrelated to participation in the study. Table 1 presents baseline demographic and clinical characteristics of the participants. At baseline, the DBT and TAU groups did not differ in demographics, education, employment, diagnoses, medication use, EQ-5D utility, and SIDAS suicidal ideation.

**Table 1:** Baseline characteristics of participants by condition

	DBT (*n* = 63)	TAU (*n* = 60)	All (*n* = 123)
Age in years, M (SD)	36.9 (10.6)	37.9 (12.1)	37.4 (11.3)
Gender, *n* (%)			
Male	33 (52)	31 (52)	64 (52)
Female	30 (48)	28 (47)	58 (47)
Non-binary	0 (0)	1 (2)	1 (1)
Non-Dutch origin, *n* (%)	2 (3)	1 (2)	3 (2)
Living conditions, *n* (%)			
Married or cohabitating	20 (32)	15 (25)	35 (28)
Children	18 (29)	11 (18)	29 (24)
Alone	24 (38)	30 (50)	54 (44)
Sheltered housing	6 (10)	7 (12)	13 (11)
Employed, *n* (%)	20 (32)	23 (38)	43 (35)
Education, *n* (%)			
University	8 (13)	6 (10)	14 (11)
Higher vocational	4 (6)	8 (13)	12 (10)
Middle vocational	22 (35)	19 (32)	41 (33)
Lower vocational	2 (3)	0 (0)	2 (2)
Secondary	21 (35)	22 (37)	43 (35)
DSM-5 diagnosis			
ASD, *n* (%)	63 (100)	60 (100)	123 (100)
Age at diagnosis of ASD, M (SD)	29.2 (12.7)	30.5 (14.3)	29.8 (13.4)
Number of diagnoses, M (SD)	2.0 (0.7)	1.8 (0.7)	1.9 (0.7)
Prior DSM diagnoses^a^ (%)	54 (86)	46 (77)	81 (100)
DSM comorbidity			
Depressive disorder n (%)	28 (44)	20 (33)	48 (39)
Personality disorder n (%)	8 (10)	4 (7)	12 (10)
Post-traumatic stress disorder	5 (8)	4 (7)	9 (7)
Anxiety disorder n (%)	2 (3)	4 (7)	6 (5)
Obsessive compulsive disorder n (%)	4 (6)	3 (5)	7 (6)
Attention deficit hyperactivity disorder n (%)	14 (22)	15 (25)	29 (24)
Bipolar disorder n (%)	1 (2)	-	1 (1)
Eating disorder n (%)	4 (6)	4 (7)	8 (7)
Substance-related disorder n (%)	7 (11)	4 (7)	11 (9)
Medication use^b^, n (%)			
Antidepressants	36 (57)	28 (47)	64 (52)
Antipsychotics	23 (37)	21 (35)	44 (36)
ADHD	10 (16)	9 (15)	19 (15)
Outcome			
SIDAS suicidal ideation (SD)	33.5 (8.9)	32.4 (8.8)	33.0 (8.9)

*DBT* dialectical behaviour therapy, *TAU* treatment as usual, *n* number of participants, *M* = mean, *SD* standard deviation, *DSM-5* Diagnostic and Statistical Manual of Mental Disorders, Fifth Edition, *ASD* Autism Spectrum Disorder, *ADHD* Attention Deficit Hyperactivity Disorder, *SIDAS* Suicidal Ideation Attributes Scale

^a^Prior *DSM diagnoses* are available on request

^b^Which and dosage antidepressants, antipsychotics, and ADHD medication are available on request

### Resource use

Table 2 describes participants'healthcare service use over time. At t_12_, healthcare use was notably lower for DBT than for TAU, particularly regarding hospital admissions, psychotherapy and psychiatry visits, and psychiatric nurse visits.

**Table 2:** Total healthcare utilisation and days of absenteeism and presenteeism in the TAU and DBT over time

	*Baseline t*_*0*_		*Post-treatment t*_*6*_		*Follow-up t*_*12*_	
Resource use	TAU (*n* = 60)	DBT (*n* = 63)	TAU (*n* = 54)	DBT (*n* = 56)	TAU (*n* = 54)	DBT (*n* = 54)
Consult general practitioner	60	88	68	75	78	69
Home visit general practitioner	0	0	0	0	1	1
Contact GP’s assistant	7	4	10	5	9	2
Outpatient session psychotherapist/psychiatrist	377	460	618	829	540	87
Home visit psychotherapist/psychiatrist	17	21	45	0	3	0
Contact psychiatric nurse	94	62	162	66	171	90
Contact specialised nurse	10	57	7	5	12	7
Day inpatient mental healthcare	89	130	80	10	190	7
Psychiatric day treatment	8	0	0	0	0	0
Contact social worker	69	94	118	159	143	119
Contact psychomotor therapy	14	14	5	13	16	13
Contact physiotherapist	39	64	60	80	118	76
Visit home care	72	58	36	60	67	36
Day in general hospital care	23	33	23	19	23	36
Visit of emergency care	7	4	11	2	12	3
Contact religious healer	3	9	3	6	4	15
Session Self-help group	16	32	0	2	14	8
Hour help from family and friends	221	279	273	222	207	269
Lost workday absenteeism	380	304	210	261	199	194
Lost workday presenteeism	218	332	105	210	89	216

*DBT* dialectical behaviour therapy, *TAU* treatment as usual, *n* number of participants, *GP* general practitioner

### Costs

Table 3 describes the costs in both groups at t_0_, t_6_, and t_12_ as seen from both the healthcare and societal perspectives, plus the cumulative costs over the full 12-month period. Regarding the cumulative healthcare costs, the DBT group incurred €11,218 per participant, while the TAU group had costs of €11,590. The per-participant cumulative societal costs were €17,454 in DBT and €17,222 in TAU on average.

**Table 3:** Average per-participant costs per 3-month period in Euros for the year 2023 by time and condition

	*t*_*0*_		*t*_*6*_		*t*_*12*_		*Cumulative costs* (€)	
	TAU	DBT	TAU	DBT	TAU	DBT	TAU	DBT
Costs (€), mean (SD)	(*n* = 60)	(*n* = 63)	(*n* = 54)	(*n* = 56)	(*n* = 54)	(*n* = 54)		
Healthcare costs	1910 (2115)	2253 (3177)	2934 (2101)	2908 (1512)	3485 (5482)	3057 (1416)	11,590 (7381)	11,218 (5616)
Patient and family costs	84 (97)	105 (109)	101 (101)	69 (85)	89 (101)	98 (100)		
Productivity costs	1925 (4290)	1828 (4280)	1179 (3816)	1504 (5038)	1126 (3678)	1071 (3411)		
Societal costs	3918 (5009)	4153 (5308)	4238 (4797)	4482 (5597)	5723 (6612)	4227 (3755)	17,222 (14,437)	17,454 (16,896)

Total annual costs per patient were calculated using the area-under-the-curve (AUC) method, with linear interpolation between baseline, 6-month, and 12-months assessment. Reported mean costs in this table are based on non-imputed data; cumulative costs were calculated using imputed data (*n* = 60 in TAU, *n* = 63 in DBT). *SD* standard deviation, *DBT* dialectical behaviour therapy, *TAU* treatment as usual, *t*_*0*_ baseline, *t*_*6*_ post-treatment, *t*_*12*_ 12-month follow-up assessment

### Effects

#### EQ-5D utility and QALY

The average utility in DBT increased from 0.50 (95%CI: 0.434 to 0.585) at baseline to 0.79 (95%CI: 0.745 to 0.844) at t_6,_ rising to 0.81 (95%CI: 0.778 to 0.859) at t_12_. In the TAU group, average utility increased from 0.45 (95%CI: 0.384 to 0.535) at baseline to 0.57 (95%CI: 0.496 to 0.660) at t_6_ and 0.59 (95%CI: 0.514 to 0.670) at t_12_, indicating an overall improvement in health-related quality of life over time for both conditions. However, the DBT group demonstrated a significantly greater improvement in QALYs, with a calculated difference of 0.18 (95% CI: 0.11 to 0.26, z = 5.01, *p* < 0.001), indicating a greater QALY gain in the DBT group (QALYs = 0.728, 95% CI: 0.685 to 0.770) compared to the TAU group (QALYs = 0.544, 95% CI: 0.483 to 0.605).

#### SIDAS treatment response

In the DBT group, 36 out of 63 participants (57%) showed a treatment response defined as a reduction in suicidal ideation of at least 50% between t_0_ and t_12_ and reaching a SIDAS score below 20 at t_12_. In the TAU group, 24 out of 60 participants (40%) showed treatment response. The statistical analysis revealed a difference in response rates of 0.17, and this difference was not significant (z = 1.92, *p* = 0.055, 95%CI: −0.004 to 0.346).

### Incremental costs per QALY

#### Healthcare perspective

**Fig. 1:**
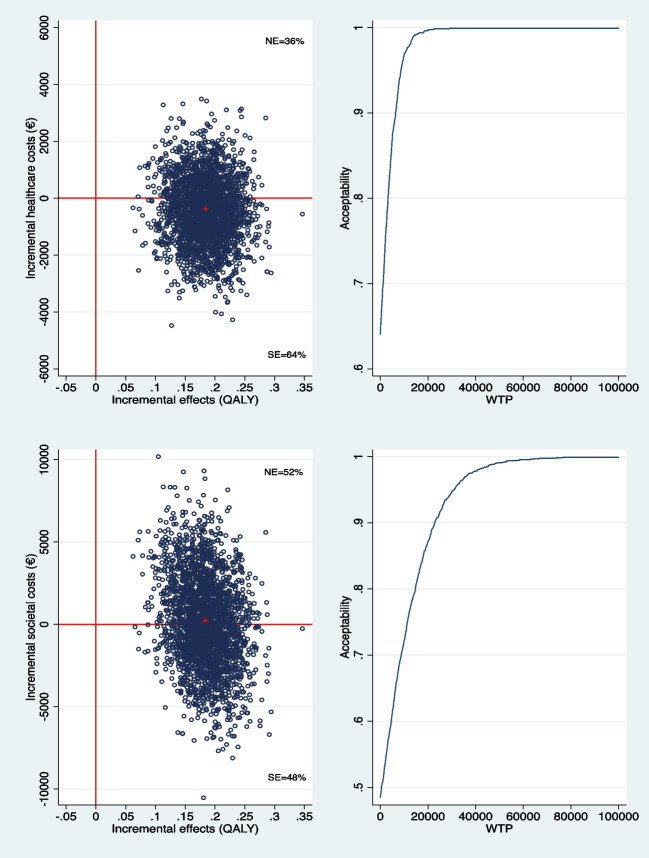
Healthcare (upper panel) and Societal (lower panel) costs in € per QALY: Cost-effectiveness plane (left panel) and acceptability curve (2500 bootstraps) (right panel). ICER plane quadrants: NE, Northeast (more effective, more expensive); SE, Southeast (the dominant quadrant: more effective, less expensive) WTP: willingness to pay

As seen from the healthcare perspective, DBT was associated with lower incremental healthcare costs of –€371 (bootstrap 95%CI: –€2,645 to €1,902) and gained 0.184 QALYs (bootstrap 95%CI: 0.111 to 0.255) compared to TAU. As can be seen in the upper panels of Fig. 1, DBT had a 64% likelihood to gain more QALYs for less costs than TAU. In other words, DBT had an 64% likelihood to be the ‘dominant’ (i.e. the preferred) treatment option.

#### Societal perspective

From a societal perspective, DBT was associated with incremental costs of €232 (bootstrap 95%CI: –€5,162 to 5,626) and a gain of 0.184 QALYs (bootstrap 95%CI: 0.111 to 0.255) compared to TAU. The cost-effectiveness analysis indicated that 52% of the incremental ICERs were in the northeast quadrant of the cost-effectiveness plane, suggesting that DBT is likely to be more effective than TAU, albeit at higher cost. Since the extra cost of €232 per QALY is well below the willingness-to-pay ceiling of €50,000 per QALY, DBT can still be regarded as cost-effective.

## Incremental costs per treatment responder

From the healthcare perspective, DBT showed dominance with less costs by €371 (bootstrap 95%CI: –€2,645 to €1,902) and better treatment response by 0.17 (bootstrap 95%CI: –0.003 to 0.346). When viewed from the societal perspective, DBT is associated with an average increase in cost of €234 (bootstrap 95%CI: –€5,162 to 5,626) and improved treatment response by 0.17 (bootstrap 95%CI: –0.003 to 0.346). The cost-effectiveness and cost-utility analysis of the base case analysis and the sensitivity analyses are presented in Table 4 in a compact format.

**Table 4:** Results of base-case analysis and sensitivity analysis (bootstraps = 2500)

*Analysis*							
Perspective	Incremental costs, €	Incremental effects	ICER cost/effect, €	ICER plane quadrants, %			
Outcome				NE	NW	SW	SE
*Base-case*							
Healthcare							
QALY	–371	0.184	dominant^1^	36	0	0	64
Response	–371	0.171	dominant^1^	35	1	2	62
Societal							
QALY	231	0.184	costeffective^2^	52	0	0	48
Response	231	0.171	costeffective^2^	50	2	1	47
*Sensitivity*							
Healthcare							
QALY	–367	0.165	dominant^1^	37	0	0	63
Response	–367	0.076	dominant^1^	30	7	11	52
Societal							
QALY	87	0.165	dominant^1^	49	0	0	51
Response	87	0.076	-	38	12	7	43

*ICER*incremental cost-effectiveness ratio, *QALY*quality adjusted life year, *ICER*plane quadrants are*NE*Northeast (more effective, more expensive), *NW*Northwest (less effective, more expensive), *SW*Southwest (less effective, less expensive), and*SE*Southeast (the dominant quadrant: more effective, less expensive)

^1^“Dominant”, because dialectical behaviour therapy costs less than treatment as usual (TAU) and has better effectiveness than TAU

^2^“Cost-effective” because much lower than the €50.000/QALY WTP ceiling

### Sensitivity analysis

Sensitivity analysis confirmed base-case results with LOCF-imputed data (Table 4). From the healthcare perspective, DBT had a 64% likelihood to be dominant given its QALY gain of 0.165 (bootstrap 95%CI: 0.085 to 0.244) and a reduction in healthcare costs of –€367 (bootstrap 95%CI: –€2,844 to 2,110). Viewed from the societal perspective, DBT incurred an increase in cost of €87 (bootstrap 95%CI: –€5,616 to €5,719) and gained 0.165 QALYs (bootstrap 95%CI: 0.085 to 0.244). The cost-effectiveness analysis indicated that 52% of the simulated ICERs occurred in the northeast quadrant, suggesting a"pay more, get more"scenario for DBT. This indicates that DBT provides substantial benefits, as evidenced by a treatment response gain of 0.165 QALYs (bootstrap 95%CI: 0.085 to 0.244), underscoring its potential value despite higher costs.

All in all, the sensitivity analyses are consistent with the base-case findings.

## Discussion

### Principal findings

We evaluated the cost-effectiveness and cost-utility of a time-limited DBT intervention compared to TAU for autistic individuals with suicidal behaviours alongside an RCT. From the healthcare perspective, the analysis showed a 64% likelihood of DBT being dominant, providing more health gains (in terms of QALYs and better treatment response rates) for lower costs. From a societal perspective, DBT demonstrates increased benefits at higher costs, with 52% of ICER points in the northeast quadrant. Despite the higher costs, the additional €232 per QALY is well below the €50,000 threshold, affirming DBT's cost-effectiveness. Sensitivity analyses supported the robustness of these findings, underscoring the conditional cost-effectiveness of DBT across different perspectives.

### Finding in the context

The complex nature of autism and the unique needs of autistic individuals significantly contribute to its enduring economic costs, encompassing healthcare, social services, education, and related expenses, thereby highlighting its substantial impact on affected individuals and society [8]. This study excludes educational costs as our adult participants were beyond their schooling years, in contrast to Sampaio et al., (2022) [8], which focused on younger individuals with autism and observed different economic impacts. The demographic difference alone does not fully explain why productivity losses are a minor component of economic costs in this study compared to earlier research [34, 35]. This discrepancy may be due to the absence of productivity loss assessments among the parents in the study group.

Regarding the economic evaluations, our analysis applied a willingness-to-pay (WTP) threshold of €50,000 per QALY. This figure is typical in many countries, although the Netherlands may find acceptable a WTP up to €80,000 per QALY for severe conditions.

These findings may encourage policymakers in healthcare to consider the wide-scale implementation of DBT as a cost-effective intervention for autistic individuals with suicidality. The demonstrated cost-effectiveness suggests that DBT can alleviate the health and disease burden associated with this population. Moreover, while our study was conducted in the Netherlands, the results may cautiously be generalised to other North-West European countries and Australia, where QALYs are calculated using similar EQ-5D value sets and where comparable willingness-to-pay thresholds (€50,000/£50,000/$50,000 per QALY) are often applied. This generalizability highlights the broader applicability of our findings to healthcare systems with similar economic and evaluative frameworks. Further research is needed to explore these aspects more thoroughly.

### Limitations

Our study has several limitations. First, it was not powered to statistically test cost differences due to the large standard errors associated with cost data, necessitating very large sample sizes for hypothesis testing. Instead, a probabilistic decision-making approach was adopted to make inferences about cost-effectiveness. This was done using 2,500 bootstrap replications to visualise uncertainty around the mean ICER across ICER plane quadrants and to depict acceptability curves.

Second, all costs and outcomes relied on self-report, which may have introduced errors and potential biases.

Third, using the EQ-5D-3L instead of the EQ-5D-5L may have reduced sensitivity to changes in health-related quality of life and increased ceiling effects.

Lastly, data collection was limited to 12 months post-baseline, leaving long-term effects and costs unknown. Despite this limitation, our evaluations revealed substantial cost reductions primarily due to reduced hospital admissions in the DBT group. Future research should explore DBT's longer term cost-effectiveness.

## Conclusion

DBT has emerged as a cost-effective treatment for addressing suicidality in autistic individuals compared to treatment as usual. This carries the promise that DBT could play an important role in alleviating both the health and economic burden associated with autism spectrum disorder with comorbid suicidality. Previously, effective interventions for autistic individuals at risk of suicidality were unavailable. Our findings demonstrate that DBT not only effectively reduces suicidality and improves quality of life but also does so at costs that are comparable to, or sometimes lower than, treatment as usual. Policymakers in healthcare may consider the broad implementation of DBT in the Netherlands and also in other countries with similar healthcare systems. Future research should assess DBT’s long-term impacts and its transferability to other countries and map pathways towards upscaled implementation.

## Supplementary Information 

Below is the link to the electronic supplementary material.

Supplementary file1 (DOCX 248 KB)

## Data Availability

The data on which this study was based are available from the corresponding author upon reasonable request.

## References

[1] Bai, D., Yip, B.H.K., Windham, G.C., et al.: Association of genetic and environmental factors with autism in a 5-country cohort. JAMA Psychiatry **76**(10), 1035–1043 (2019) 31314057 10.1001/jamapsychiatry.2019.1411 PMC6646998

[2] Brugha, T.S., Spiers, N., Bankart, J., et al.: Epidemiology of autism in adults across age groups and ability levels. Br. J. Psychiatry **209**(6), 498–503 (2016) 27388569 10.1192/bjp.bp.115.174649

[3] Buck, T.R., Viskochil, J., Farley, M., et al.: Psychiatric comorbidity and medication use in adults with autism spectrum disorder. J. Autism Dev. Disord. **44**(12), 3063–3071 (2014) 24958436 10.1007/s10803-014-2170-2 PMC4355011

[4] Simonoff, E., Jones, C.R., Baird, G., Pickles, A., Happé, F., Charman, T.: The persistence and stability of psychiatric problems in adolescents with autism spectrum disorders. J. Child Psychol. Psychiatry **54**(2), 186–194 (2013) 22934711 10.1111/j.1469-7610.2012.02606.x

[5] Lever, A.G., Geurts, H.M.: Psychiatric co-occurring symptoms and disorders in young, middle-aged, and older adults with autism spectrum disorder. J. Autism Dev. Disord. **46**(6), 1916–1930 (2016) 26861713 10.1007/s10803-016-2722-8 PMC4860203

[6] Mutluer, T., Aslan Genç, H., Özcan Morey, A., et al.: Population-based psychiatric comorbidity in children and adolescents with autism spectrum disorder: A meta-analysis. Front. Psychiatry **5**(13), 856208 (2022) 10.3389/fpsyt.2022.856208 PMC9186340 35693977

[7] Huntjens, A., Landlust, A., Wissenburg, S., van der Gaag, M.: The prevalence of suicidal behavior in autism spectrum disorder. Crisis **45**(2), 144–153 (2024) 37668055 10.1027/0227-5910/a000922

[8] Sampaio, F., Feldman, I., Lavelle, T.A., Skokauskas, N.: The cost-effectiveness of treatments for attention deficit-hyperactivity disorder and autism spectrum disorder in children and adolescents: a systematic review. Eur. Child Adolesc. Psychiatry **31**(11), 1655–1670 (2022) 33751229 10.1007/s00787-021-01748-z PMC9666301

[9] Rogge, N., Janssen, J.: The economic costs of autism spectrum disorder: A literature review. J. Autism Dev. Disord. **49**(7), 2873–2900 (2019) 30976961 10.1007/s10803-019-04014-z

[10] Karst, J.S., Van Hecke, A.V.: Parent and family impact of autism spectrum disorders: a review and proposed model for intervention evaluation. Clin. Child. Fam. Psychol. Rev. **15**(3), 247–277 (2012) 22869324 10.1007/s10567-012-0119-6

[11] DeCou, C.R., Comtois, K.A., Landes, S.J.: Dialectical behavior therapy is effective for the treatment of suicidal behavior: A meta-analysis. Behav. Ther. **50**(1), 60–72 (2019) 30661567 10.1016/j.beth.2018.03.009

[12] Huntjens, A., van den Bosch, L.M.C.W., Sizoo, B., Kerkhof, A., Huibers, M.J.H., van der Gaag, M.: The effect of dialectical behaviour therapy in autism spectrum patients with suicidality and/ or self-destructive behaviour (DIASS): study protocol for a multicentre randomised controlled trial. BMC Psychiatry **20**(1), 127 (2020) 32183793 10.1186/s12888-020-02531-1 PMC7079441

[13] Huntjens, A., van den Bosch, L.M.C.W., Sizoo, B., Kerkhof, A., Smit, F., van der Gaag, M.: The effectiveness and safety of dialectical behavior therapy for suicidal ideation and behavior in autistic adults: a pragmatic randomized controlled trial. Psychol. Med. **4**(12), 1–12 (2024) 10.1017/S0033291724000825 38606582

[14] Linehan, M.M.: Cognitive behavioural therapy of borderline personality disorder. New York: The Guilford Press (1993)

[15] Kan, C.C., Geurts, H.M., Sizoo, B.B.: Multidisciplinaire richtlijn diagnostiek en behandeling van autismespectrumstoornissen bij volwassenen. Utrecht: De Tijdstroom (2013)

[16] Hoekstra, R.A., Vinkhuyzen, A.A., Wheelwright, S., et al.: The construction and validation of an abridged version of the autism-spectrum quotient (AQ-Short). J. Autism Dev. Disord. **41**(5), 589–596 (2011) 20697795 10.1007/s10803-010-1073-0 PMC3076581

[17] van Spijker, B.A., Batterham, P.J., Calear, A.L., et al.: The suicidal ideation attributes scale (SIDAS): Community-based validation study of a new scale for the measurement of suicidal ideation. Suicide Life-Threat. Behav. **44**(4), 408–419 (2014) 24612048 10.1111/sltb.12084

[18] Comtois, K.A., Linehan, M.M.: Lifetime parasuicide count: description and psychometrics. Proceedings from 32nd annual meeting of the American association of suicidology, Houston, TX (1999)

[19] Wechsler, D.: Wechsler abbreviated scale of intelligence, second edition (WASI-II). San Antonio, TX: (2nd ed) NCS Pearson (2011)

[20] Neacsiu, A.D., Eberle, J.W., Kramer, R., Wiesmann, T., Linehan, M.M.: Dialectical behavior therapy skills for transdiagnostic emotion dysregulation: A pilot randomized controlled trial. Behav. Res. Ther. **59**, 40–51 (2014) 24974307 10.1016/j.brat.2014.05.005

[21] Anderson, S., Morris, J.: Cognitive behaviour therapy for people with Asperger syndrome. Behav. Cogn. Psychother. **34**(3), 293–303 (2006)

[22] König, H.H., Roick, C., Angermeyer, M.C.: Validity of the EQ-5D in assessing and valuing health status in patients with schizophrenic, schizotypal or delusional disorders. Eur. Psychiatry **22**(3), 177–187 (2007) 17142014 10.1016/j.eurpsy.2006.08.004

[23] Lamers, L.M., McDonnell, J., Stalmeier, P.F., Krabbe, P.F., Busschbach, J.J.: The Dutch tariff: Results and arguments for an effective design for national EQ-5D valuation studies. Health Econ. **15**(10), 1121–1132 (2006) 16786549 10.1002/hec.1124

[24] Bouwmans, C., De Jong, K., Timman, R., et al.: Feasibility, reliability and validity of a questionnaire on healthcare consumption and productivity loss in patients with a psychiatric disorder (TiC-P). BMC Health Serv. Res. **13**, 217 (2013) 23768141 10.1186/1472-6963-13-217 PMC3694473

[25] van den Brink, M., van den Hout, W.B., Stiggelbout, A.M., Putter, H., van de Velde, C.J., Kievit, J.: Self-reports of health-care utilization: diary or questionnaire. Int. J. Technol. Assess. Health Care **21**(3), 298–304 (2005) 16110708 10.1017/s0266462305050397

[26] Hakkaart - van Roijen, L.P.S., Kanters, T.: Kostenhandleiding voor economische evaluaties in de gezondheidszorg: Methodologie en referentieprijzen. (Herziene versie 2024 ed) (2024)

[27] Farmacotherapeutisch-Kompas. (Pharmaceutical Compass). URL:https://www.farmacotherapeutischkompas.nl/ (2023). Accessed 2023–10–23

[28] Kanters, T.A., Bouwmans, C.A.M., van der Linden, N., Tan, S.S., Hakkaart-van, R.L.: Update of the Dutch manual for costing studies in health care. PLoS One **12**(11), e0187477 (2017) 29121647 10.1371/journal.pone.0187477 PMC5679627

[29] Husereau, D., Drummond, M., Augustovski, F., et al.: Correction to: Consolidated health economic evaluation reporting standards 2022 (CHEERS 2022) statement: Updated reporting guidance for health economic evaluations. Appl. Health Econ. Health Policy **20**(5), 781–782 (2022) 35840812 10.1007/s40258-022-00743-y PMC9385799

[30] OECD. Purchasing power parities (PPP) Indicator. 10.1787/1290ee5a-en. https://data.oecd.org/conversion/purchasing-power-parities-ppp.htm (2024). Accessed on 19 March 2024

[31] Demirkaya, S.K., Tutkunkardaş, M.D., Mukaddes, N.M.: Assessment of suicidality in children and adolescents with diagnosis of high functioning autism spectrum disorder in a Turkish clinical sample. Neuropsychiatr. Dis. Treat. **12**, 2921–2926 (2016) 27956832 10.2147/NDT.S118304 PMC5113915

[32] Zorginstituut, N.: Kosteneffectiviteit in de praktijk (Cost-effectiveness analysis in practice). Diemen, The Netherlands: Zorginstituut Nederland (2015)

[33] Salomon, J.A., Vos, T., Hogan, D.R., Gagnon, M., Naghavi, M.: Common values in assessing health outcomes from disease and injury: Disability weights measurement study for the global burden of disease study 2010. Lancet **380**(9859), 2129–2143 (2012) 23245605 10.1016/S0140-6736(12)61680-8 PMC10782811

[34] Buescher, A.V., Cidav, Z., Knapp, M., Mandell, D.S.: Costs of autism spectrum disorders in the United Kingdom and the United States. JAMA Pediatr. **168**(8), 721–728 (2014) 24911948 10.1001/jamapediatrics.2014.210

[35] Ganz, M.L.: The lifetime distribution of the incremental societal costs of autism. Arch. Pediatr. Adolesc. Med. **161**(4), 343–349 (2007) 17404130 10.1001/archpedi.161.4.343

