# Supplementary material for: Cost-effectiveness of dialectical behavioural therapy versus treatment as usual for autism with suicidal behaviours: single-blind randomised controlled trial
Source: Eur J Health Econ. 2025 May 31;26(9):1633–43. doi: 10.1007/s10198-025-01794-3 (PMC12618375; doi:10.1007/s10198-025-01794-3)
Supplement: Supplementary file 1 — Supplementary file1 (DOCX 248 KB) [file 10198_2025_1794_MOESM1_ESM.docx]

Online resource

**Cost-Effectiveness of Dialectical Behavioural Therapy versus Treatment as Usual for Autism with Suicidal Behaviours: single-blind randomised controlled trial**

Anne Huntjens^1,2^, Filip Smit^1,3^, L. M. C. (Wies) van den Bosch^4^, Ad Kerkhof ^1^, Bram Sizoo^5^, Mark van der Gaag^1,2^

**Author affiliations**

^1^ Department of Clinical Psychology and Amsterdam Public Mental Health Research Institute, Vrije Universiteit Amsterdam, Amsterdam, Netherlands

^2^ Department of Mark van der Gaag Research Centre, Parnassia Psychiatric Institute, The Hague, Netherlands

^3^ Department of Epidemiology and Biostatistics, Amsterdam University Medical Centers, Amsterdam, Netherlands

^4^ Independent researcher, Dialexis, Nijmegen, Netherlands

^5^ Department of Clinical Psychology, University of Amsterdam, Amsterdam, Netherlands

**Corresponding author:** Anne Huntjens; [a.huntjens@vu.nl](mailto:a.huntjens@vu.nl); +316 14433568; Department Clinical Psychology,Vrije Universiteit Amsterdam, De Boelelaan 1105, 1081 HV Amsterdam

The Netherlands

**Content**

This file contains supplementary Tables and Figures.

**Figure S1**: Flowchart depicting the progression of participants throughout the trial.

**Table S1:** Overview of the costs associated with healthcare units and productivity losses.

**Table S2**: Detailed account of travel costs.

**Table S3:** Compliance checklist for the Consolidated Health Economic Evaluation Reporting Standards (CHEERS).

**Figure S1.** The flow of participants through the trial

**44**  Excluded

- **6** did not meet inclusion criteria
- **19** unappealing patient information letter
- **4** due to randomisation
- **7** unable/ unwilling to travel to location
- **5** costs too much time
- **3** due to treatment elsewhere

**167** people assessed for eligibility

**123** Randomised *(week 0)*

Allocation

**63** Allocated to DBT *(week 1-26)*

**60** Allocated to TAU *(week 1-26)*

Post-treatment

**57** attended 6-month assessment *(week 27)*

**56** attended 6-month assessment *(week 27)*

Follow-Up

**57** attended 12-month follow-up assessment *(week 53)*

**55** attended 12-month follow-up assessment *(week 53)*

**63** included intention-to-treat group

**60** included intention-to-treat group

_DBT= dialectical behaviour therapy; TAU; treatment as usual; posttreatment:27 weeks post-baseline, which was after completion of the intervention; follow-up: 53 weeks post-baseline_

**Table S1. Unit cost price in Euro for the year 2023**

| Health care unit | Unit cost price, € | Reference |  |
| --- | --- | --- | --- |
| General practitioner, visit | 43.31 | 1 |  |
| General practitioner, home visit | 48.21 | 1 |  |
| Gp’s assistant, contact | 20.87 | 1 |  |
| Outpatient psychotherapist/psychiatrist, session | 134.00 | 1 |  |
| Psychotherapist/psychiatrist, home visit | 201.00 | 2 |  |
| Psychiatric nurse, contact | 28.50 | 1 |  |
| Specialized nurse, contact | 28.90 | 1 |  |
| Inpatient mental healthcare, day | 327.00 | 1 |  |
| Social worker, contact | 127.00 | 1 |  |
| Psychomotor therapy, session | 42.91 | 1 |  |
| Physiotherapist, contact | 38.89 | 1 |  |
| Home care, home visit | 64.00 | 1 |  |
| General hospital, day | 644.00 | 1 |  |
| Emergency care, visit | 258.00 | 1 |  |
| Religious healer, contact | 18.80 | 3 |  |
| Self-help group, session | 18.80 | 3 |  |
| Pharmacist dispensing costs | 7.00 | 1 |  |
| Anti-depressants / anxiolytics, daily dose | 0.13 | 4 |  |
| Adhd medication, daily dose | 1.13 | 4 |  |
| Antipsychotics, daily dose | 0.34 | 4 |  |
| Paid work, hour | 39.88 | 1 |  |
| Voluntary work / informal care, hour | 18.80 | 1 |  |
| _1) Hakkaart van Roijen l, Peters S, Kanters T. Kostenhandleiding voor economische evaluaties in de gezondheidszorg: methodologie en referentieprijzen, herziene versie 2024._  _2) assumed 1.5 * €134 (for outpatient psychotherapy session)_  _3) valued as €18.80 (voluntary work / informal care)_  _4) farmacotherapeutisch-kompas. (2023). Url:https://www.farmacotherapeutischkompas.nl/ [accessed 2023-10-23]_ | | | |

**Table S2. Travel costs from a random address to the nearest health service in 2023, in Euro**^1^

| Health care service | Price (kilometers return trip) |
| --- | --- |
| General practice | €0.42 (2.0 km) |
| Pharmacist | €0.50 (2.4 km) |
| Mental out-patient care | €2,94 (14 km) |
| Physiotherapist | €0,92 (4.4 km) |

_1) Hakkaart van Roijen l, Peters S, Kanters T. Kostenhandleiding voor economische evaluaties in de gezondheidszorg: methodologie en referentieprijzen, herziene versie 2024._
